# Clinical significance of local control of primary tumour in definitive radiotherapy for scalp angiosarcomas

**DOI:** 10.1111/srt.13243

**Published:** 2022-11-20

**Authors:** Tairo Kashihara, Dai Ogata, Kae Okuma, Satoshi Nakamura, Hiroki Nakayama, Taisuke Mori, Akira Takahashi, Kenjiro Namikawa, Ayaka Takahashi, Kana Takahashi, Tomoya Kaneda, Koji Inaba, Naoya Murakami, Hiroyuki Okamoto, Yuko Nakayama, Naoya Yamazaki, Hiroshi Igaki

**Affiliations:** ^1^ Department of Radiation Oncology National Cancer Center Hospital Chuo‐ku Tokyo Japan; ^2^ Department of Dermatologic Oncology National Cancer Center Hospital Chuo‐ku Tokyo Japan; ^3^ Department of Pathology and Clinical Laboratories National Cancer Center Hospital Chuo‐ku Tokyo Japan

**Keywords:** angiosarcoma, intensity‐modulated radiotherapy, radiotherapy, skin neoplasms, volumetric modulated‐arc therapy

## Abstract

**Introduction:**

Scalp angiosarcoma is a rare and aggressive cancer. Definitive radiotherapy is a treatment option for localised scalp angiosarcoma patients. Although definitive surgical resection reportedly prolongs overall survival (OS), whether initial local treatment effect affects OS when definitive radiotherapy is administered is unclear. Therefore, this study analysed whether local recurrence within 6 months of irradiation correlates with OS and cancer‐specific survival (CSS). Furthermore, how local control affects patients' quality of life was investigated.

**Materials and methods:**

Thirty‐one localised scalp angiosarcoma patients who had received definitive radiotherapy at our institution between October 2010 and July 2021 were analysed retrospectively. The most commonly used dose fractionation was 70 Gy in 35 fractions (83.9%). Local recurrence within 6 months of radiotherapy and other clinical factors were examined in univariate and subsequent multivariate analyses for correlation with OS and CSS.

**Results:**

The median follow‐up period was 16 months (range, 6–45 months). Local recurrence was detected in 16 patients (51.6%), 12 of whom had recurrence within 6 months. In multivariate analyses, the presence of local recurrence within 6 months of radiotherapy was significantly associated with OS and CSS (*p* = 0.003, 0.0001, respectively). Ten of the 16 patients with local recurrence had severe symptoms such as bleeding, pain, difficulty opening the eye and malodour.

**Conclusions:**

The initial local treatment effect was significantly associated with OS and CSS after definitive radiotherapy. Furthermore, local recurrence after radiotherapy resulted in a variety of symptoms, including bleeding and pain, which reduced the patient's quality of life.

**Abbreviations**
BNCT: boron neutron capture therapy
CSS: cause‐specific survival
CTV: clinical target volume
IMRT: intensity‐modulated radiation therapy
MFW: malignant fungating wounds
OS: overall survival
VMAT: volumetric‐modulated arc therapy

## 1 INTRODUCTION

Cutaneous angiosarcoma is a rare and aggressive cancer.^1^, ^2^, ^3^ Surgery and radiotherapy with or without chemotherapy are definitive treatment options, but prognoses are quite poor.^4^, ^5^ In a systematic review and meta‐analysis,^6^ definitive surgery was associated with improved overall survival (OS). However, some patients with locally advanced disease have unresectable lesions or refuse to undergo surgery because of concerns about postoperative cosmetic aspects, and approximately 20% of the patients receive definitive radiotherapy/chemoradiotherapy without surgery. However, whether local control after definitive radiotherapy for scalp angiosarcomas affects the OS and cause‐specific survival (CSS) is unclear. This study aimed to investigate whether the initial treatment effect of primary tumours after definitive radiotherapy for scalp angiosarcoma is a surrogate for OS and CSS using definitive radiotherapy dataset in our institution.

## 2 MATERIALS AND METHODS

### 2.1 Patient and treatment characteristics

Patient and treatment characteristics are shown in Table 1. Thirty‐five localised scalp angiosarcoma patients who had received definitive radiotherapy at our institution between October 2010 and July 2021 were retrieved. Four patients who died within 6 months of completing radiotherapy were excluded and a final analysis was conducted on 31 patients. Concurrent chemotherapy was administered to 23 patients (74.2%), and the most commonly used dose fractionation was 70 Gy in 35 fractions (83.9%). Intensity‐modulated radiotherapy or volumetric modulated arc therapy (VMAT) was delivered to 28 patients (90.3%), electron beam radiotherapy was used in two patients (6.5%), and high‐dose‐rate brachytherapy using a surface mold applicator was used in one patient (3.2%). Intentional internal high‐dose VMAT was applied for prominent lesions in three patients (9.7%).^7^

**TABLE 1:** Patient and treatment characteristics

Parameters	
Age, median (range)	75 (46‐89)
Sex	
Male	24/31 (77.4%)
Female	7/31 (22.6%)
Zubrod Performance status	
0	17/31 (54.8%)
1	11/31 (35.5%)
2	3/31 (9.7%)
Tumour invasion to face	13/31 (41.9%)
Tumour bleeding at baseline	5/31 (16.1%)
Skip lesions	26/31 (83.9%)
Nodule lesion	13/31 (41.9%)
Concurrent administration of chemotherapy	
Chemoradiotherapy, number	23/31 (74.2%)
Radiotherapy alone, number	8/31 (25.8%)
Prescription radiation dose	
70 Gy in 35 fractions	26/31 (83.9%)
66 Gy in 22 fractions	1/31 (3.2%)
60 Gy in 30 fractions	3/31 (9.7%)
51 Gy in 17 fractions	1/31 (3.2%)
Clinical target volume (cc), median (range)	268 (96.1‐611)

### 2.2 Assessment of outcomes

Treatment outcomes were evaluated by one dermatologist (plus one radiation oncologist) at least every 1–6 months. OS and CSS were investigated as outcomes. Local recurrence was defined as recurrence within the planning target volume. Considering that it may be difficult to determine the therapeutic effect for several months due to prolonged dermatitis, we analysed the correlation between the presence of local recurrence within 6 months and OS and CSS. The treatment outcomes were assessed in June 2022. Deaths other than those from angiosarcoma were treated as a competing event in the CSS. To exclude immortality time bias, a landmark analysis was performed using 6 months after completion of radiotherapy as the starting date for the survival calculation. Furthermore, the symptoms caused by the primary tumour were investigated in terms of changes in symptoms before and after treatment and at the time of recurrence. It was investigated whether the following factors were associated with OS and CSS: age, sex, Zubrod performance status, tumour invasion to face, tumour bleeding at baseline, skip lesions, nodule lesions, concurrent administration of chemotherapy, prescription radiation dose, and clinical target volume and local recurrence within 6 months.

### 2.3 Statistical analyses

The predictive factors of OS and CSS were investigated using Cox proportional hazards regression models and the Fine and Gray models, respectively. The median value was used to divide the patients into two groups. Factors with *p*‐values <0.05 in the univariate analyses were included in the multivariate analyses. Statistical significance of the multivariate analyses was set at *p* < 0.05. Statistical analyses were performed using the IBM SPSS version 26 software (IBM Corp., Armonk, NY, USA) and EZR.

### 2.4 Ethical approval

For all research involving human participants/data, written informed consent to participate in the study was obtained from participants. All analyses involving human participants performed in this study were approved by the review board of the committee of our institution (approval number: 2017–091) and were in accordance with the ethical standards of the committee and the 1964 Helsinki Declaration and its later amendments.

## 3 RESULTS

### 3.1 Survival analysis

**FIGURE 1:**
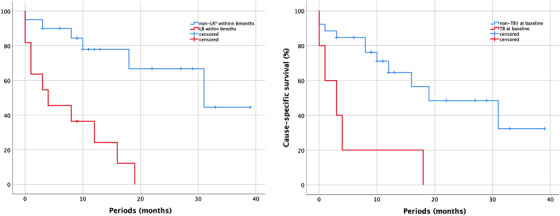
Kaplan–Meier curves comparing overall survival according to the presence or absence of local recurrence within 6 months after radiotherapy and tumour bleeding at baseline. LR*, local recurrence; TB†, tumour bleeding at baseline

The median follow‐up period was 16 months (range, 6–45 months). During the follow‐up period, 16 patients (51.6%) died: 15 from angiosarcoma and one from exacerbation of interstitial pneumonia. Local recurrence was detected in 16 patients (51.6%), 12 of whom had recurrence within 6 months. The univariate and multivariate analyses of the predictive factors of OS and CSS were shown in Table 2. In the univariate analyses, local recurrence within 6 months and tumour bleeding at baseline were significant prognostic factors. Multivariate analyses including these factors revealed that local recurrence within 6 months Hazard ratio (HR): 5.630, 95% Confidence Interval (CI): 1.822–17.396, *p* = 0.003) and tumour bleeding at baseline (HR: 3.889, 95% CI: 1.174–12.884, *p* = 0.026) were significant unfavourable factors of OS. Moreover, these factors were also significantly associated with CSS (Table 2). The Kaplan–Meier curves for OS according to the presence or absence of local recurrence within 6 months after radiotherapy and tumour bleeding at baseline are shown in Figure 1.

**TABLE 2:** The univariate and multivariate analyses of overall survival and cause‐specific survival

Parameters	
OS^*^	CSS^†^
HR (95% CI), *p* value	HR (95% CI), *p* value
**Univariate analyses**	
Age ≥ 76	
0.831 (0.301–2.298), *p* = 0.722	0.967 (0.354–2.639), *p* = 0.95
Sex (vs. Female)	
0.487 (0.166–1.433), *p* = 0.191	0.734 (0.245–2.198), *p* = 0.58
Zubrod performance status (vs. 1–2)	
0.745 (0.270–2.060), *p* = 0.571	0.574 (0.193–1.704), *p* = 0.32
Tumour invasion to face	
2.182 (0.798–5.970), *p* = 0.129	1.714 (0.633–4.644), *p* = 0.29
Tumour bleeding at baseline	
4.606 (1.508–14.065), *p* = 0.007	5.386 (1.832–15.830), *p* = 0.002
Skip lesions	
0.514 (0.160–1.652), *p* = 0.264	0.452 (0.168–1.219), *p* = 0.12
Nodule lesions	
2.396 (0.857–6.705), *p* = 0.096	2.898 (0.969–8.665), *p* = 0.057
Concurrent administration of chemotherapy	
0.549 (0.187–1.613), *p* = 0.276	0.815 (0.267–2.485), *p* = 0.72
Total biological effective dose ≥ 80 Gy	
0.558 (0.192–1.620), *p* = 0.283	0.486 (0.194–1.220), *p =* 0.12
Clinical target volume ≥ 260 cc	
2.763 (0.982–7.770), *p* = 0.054	2.268 (0.859–5.985), *p* = 0.098
Local recurrence within 6 months	
6.032 (2.021–18.004), *p* = 0.001	7.990 (2.711–23.55), *p* = 0.0001
Multivariate analyses	
Tumour bleeding at baseline	
3.889 (1.174–12.884), *p* = 0.026	4.911 (1.924–12.540), *p* = 0.0009
Local recurrence within 6 months	
5.630 (1.822–17.396), *p* = 0.003	7.819 (2.778–22.010), *p* = 0.0001

*Note*: CSS^†^; cause‐specific survival; OS^*^, overall survival.

### 3.2 Symptoms caused by primary tumours

Bleeding from the primary tumour was present in five patients (16.1%) at the initiation of radiotherapy. Haemostasis was achieved by shrinkage of the tumour in the four patients, but one of them experienced bleeding again as the tumour re‐grew. In the other patient, local control was not achieved and bleeding continued. Ten of the 16 patients with local recurrence had severe symptoms: six had tumour haemorrhage, six had facial oedema, five had severe pain, five had difficulty opening their eyes due to eyelid oedema, three had malodour, one had skin and oral ulceration due to tumour invasion, one had obstruction of the external auditory canal due to tumour, and one had septic shock due to an infection from malignant fungating wounds (MFWs). After local recurrence, three patients received palliative re‐irradiation alone, and two others received re‐irradiation after palliative surgery. Boron neutron capture therapy (BNCT) was used in one patient who received re‐irradiation.

## 4 DISCUSSION

To date, it has been unclear whether local control with definitive radiotherapy has an impact on survival in scalp angiosarcoma patients. In this study, the initial local treatment effect significantly correlated with OS and CSS. In a previous report from our institution,^8^ surgery with curative intent was significantly associated with prolonged OS. Furthermore, in a systematic review and meta‐analysis, Zayed et al. also reported that OS was significantly longer in the group that underwent definitive surgical resection.^6^ Surgery, like radiotherapy, is a local treatment, and a large resection of the primary tumour would lead to increased local control. Therefore, the results of these preceding studies that surgery prolongs OS support the findings of this study that local control prolongs OS.

In five patients who had problems with bleeding from MFWs of the primary tumour before the start of irradiation, haemostasis was achieved with local control by radiotherapy. In contrast, patients with local recurrence experienced not only bleeding but also severe pain precipitating opioid use, oedema of the face and infection. These are the only the symptoms that could be confirmed on the medical record retrospectively, and it is possible that even more patients experienced these symptoms and other psychological, cosmetic, or other distresses. An integrative review provided strong evidence that patients with MFWs suffer from multiple symptoms, such as pain, odour, exudate, bleeding, pruritus, perceived wound status and lymphedema.^9^ It has been reported that MFWs cause significant physical and psychological distress and reduce patients’ quality of life.^10^, ^11^, ^12^, ^13^ Therefore, local control of the primary tumour by radiotherapy is important to improve the quality of life of patients.

As noted in a previous study, a simple increase in the radiation dose is not realistic because many patients (66.7%) developed severe dermatitis of grade 3 or higher.^14^ Meanwhile, in a report of two cases of scalp angiosarcoma treated with BNCT, a complete response was achieved with only the appearance of grade 1 dermatitis approximately 1–2 weeks after treatment.^15^ This treatment could be important for scalp angiosarcoma to increase local control and survival rates without increasing adverse events.

Nonetheless, our study had a limitation. This was a retrospective study and may have included some bias. As mentioned above, this study might have underestimated patient symptoms. Future prospective studies are warranted to rigorously evaluate these symptoms.

## 5 CONCLUSION

In localised scalp angiosarcoma patients, local recurrence within 6 months of radiotherapy and tumour bleeding at baseline were significantly associated with OS and CSS. Moreover, local recurrence after radiotherapy resulted in a variety of symptoms, including bleeding and pain, which reduces the patient's quality of life.

## CONFLICT OF INTEREST

Dr. Igaki reports honoraria and research grant from Cancer Intelligence Care Systems, Inc., outside the submitted work. The other authors declare that they have no known competing financial interests or personal relationships that could have appeared to influence the work reported in this paper.

## FUNDING INFORMATION

This research received no specific grants from any funding agency in the public, commercial or not‐for‐profit sectors.

## Data Availability

The data that support the findings of this study are available upon request from the corresponding author. The data are not publicly available due to privacy or ethical restrictions.
